# Biopsy diagnosis of type 1 autoimmune pancreatitis: Does it bring a conclusion or confusion?

**DOI:** 10.1002/deo2.82

**Published:** 2021-12-07

**Authors:** Kenji Notohara

**Affiliations:** ^1^ Department of Anatomic Pathology Kurashiki Central Hospital Okayama Japan

**Keywords:** acinar ductal metaplasia, autoimmune pancreatitis, endoscopic ultrasound‐guided fine‐needle biopsy, observer variation, pathologist

## Abstract

A biopsy‐based diagnosis of type 1 autoimmune pancreatitis (AIP) is now feasible via an endoscopic ultrasound‐guided fine‐needle biopsy, but there are potential issues to address. The benefits of acquiring large tissue samples include more successful immunostaining for Immunoglobulin G4 and more identifications of storiform fibrosis, obliterative phlebitis, and the ductal lesions of type 1 AIP. However, storiform fibrosis may not be present in all the type 1 AIP lesions. An interobserver agreement study revealed only slight‐to‐moderate agreement among pathologists diagnosing the histological findings of type 1 AIP. Potential reasons for disagreement are the different time phases of the inflammation (which result in heterogeneous histological pictures), a focal appearance of the typical histological findings, and the different definitions used by pathologists. We have thus devised guidance for diagnosing type 1 AIP based on biopsy tissues. In this guidance, we define each histological finding of type 1 AIP, for example, storiform fibrosis as a swirling arrangement of inflammatory cells, spindle‐shaped cells, and delicate collagens as a unit. The necessity of elastic stains for identifying obliterative phlebitis is explained, with examples of mimickers. Another important purpose of a biopsy in type 1 AIP cases is differentiation from pancreatic ductal adenocarcinoma (PDAC). In this situation, acinar‐ductal metaplasia observed in type 1 AIP is a mimicker of PDAC and should not be confused. For the resolution of potential disagreements among pathologists, a multi‐disciplinary approach with the collaboration of clinicians, radiologists, and pathologists is necessary to avoid confusion.

## INTRODUCTION

Autoimmune pancreatitis (AIP) is a unique inflammatory disorder of putative autoimmune etiology in the pancreas. Tumefaction is characteristic of AIP, and the differentiation of AIP from pancreatic ductal adenocarcinoma (PDAC) is crucial. Two distinct types of AIP, types 1 and 2, are recognized.^1^, ^2^, ^3^, ^4^, ^5^, ^6^, ^7^, ^8^, ^9^, ^10^ Type 1 AIP is a pancreatic manifestation of Immunoglobulin G4 (IgG4)‐related disease, but type 2 is not.^11^ Type 1 AIP can be diagnosed based on the clinical, radiological, and histological findings according to the International Consensus Diagnostic Criteria (ICDC)^5^ or the Japan Pancreas Society Clinical Diagnostic Criteria 2018 of AIP (JPS2018).^12^ In type 2 AIP, a histological identification of granulocytic epithelial lesion (GEL) in the pancreatic ducts is mandatory to confirm the diagnosis, because type 2 AIP lacks specific clinical findings and surrogate markers (with the exception that some patients suffer from inflammatory bowel disease).^5^

The histological findings of type 1 AIP, which are designated as lymphoplasmacytic sclerosing pancreatitis,^1^, ^13^ are so unique that the diagnosis can be rendered based on the histological findings alone. In the ICDC,^5^ resected pancreatic specimens and endoscopic ultrasound (EUS)‐guided Tru‐Cut biopsy (EUS‐TCB) samples are regarded as suitable for diagnosing type 1 AIP. EUS‐guided fine‐needle aspiration (EUS‐FNA) specimens are too small to render the diagnosis of type 1 AIP, although they are useful for distinguishing AIP from PDAC. The acquisition of sufficient histological specimens by a EUS‐guided fine‐needle biopsy (EUS‐FNB) recently became possible, and the biopsy‐based diagnosis of type 1 AIP is thus now feasible. In this review article, I will discuss efficacy, limitations, and pitfalls in the diagnosis of type 1 AIP with biopsy specimens.

### Histological features of type 1 AIP for the biopsy diagnosis

**FIGURE 1:**
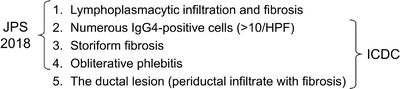
Comparison of histological items in the Japan Pancreas Society Clinical Diagnostic Criteria 2018 (JPS2018) and the International Consensus Diagnostic Criteria (ICDC) for autoimmune pancreatitis (AIP). HPF: high‐power field

Histological findings of type 1 AIP are characterized particularly by the following five features: (1) diffuse lymphoplasmacytic infiltration and fibrosis, (2) numerous IgG4‐positive cells, (3) storiform fibrosis, (4) obliterative phlebitis, and (5) the ductal lesion (periductal infiltrates with fibrosis). As items of the histological findings, features (1) through (4) are adopted in the JPS2018,^12^ whereas features (2) through (5) are used in the ICDC^5^ (Figure 1). This is based on the results of an international agreement study among pathologists using resected specimens.^14^ In that study, the kappa (κ) value of the distinction among types 1 and 2 AIP and chronic alcoholic pancreatitis was 0.08 (slight agreement) in the first round but increased to 0.54 (moderate agreement) in the second round by defining characteristic histological features, that is, features (1) through (5) for type 1 AIP and the presence of GEL for type 2.^14^ Importantly, when three or more features are identified, the diagnosis of type 1 AIP is established based on the histological findings alone in both the JPS2018 and ICDC criteria. Because features (1) and (2) are relatively easily identified, features (3) through (5) are key findings to make a definitive histological diagnosis of type 1 AIP.

**FIGURE 2:**
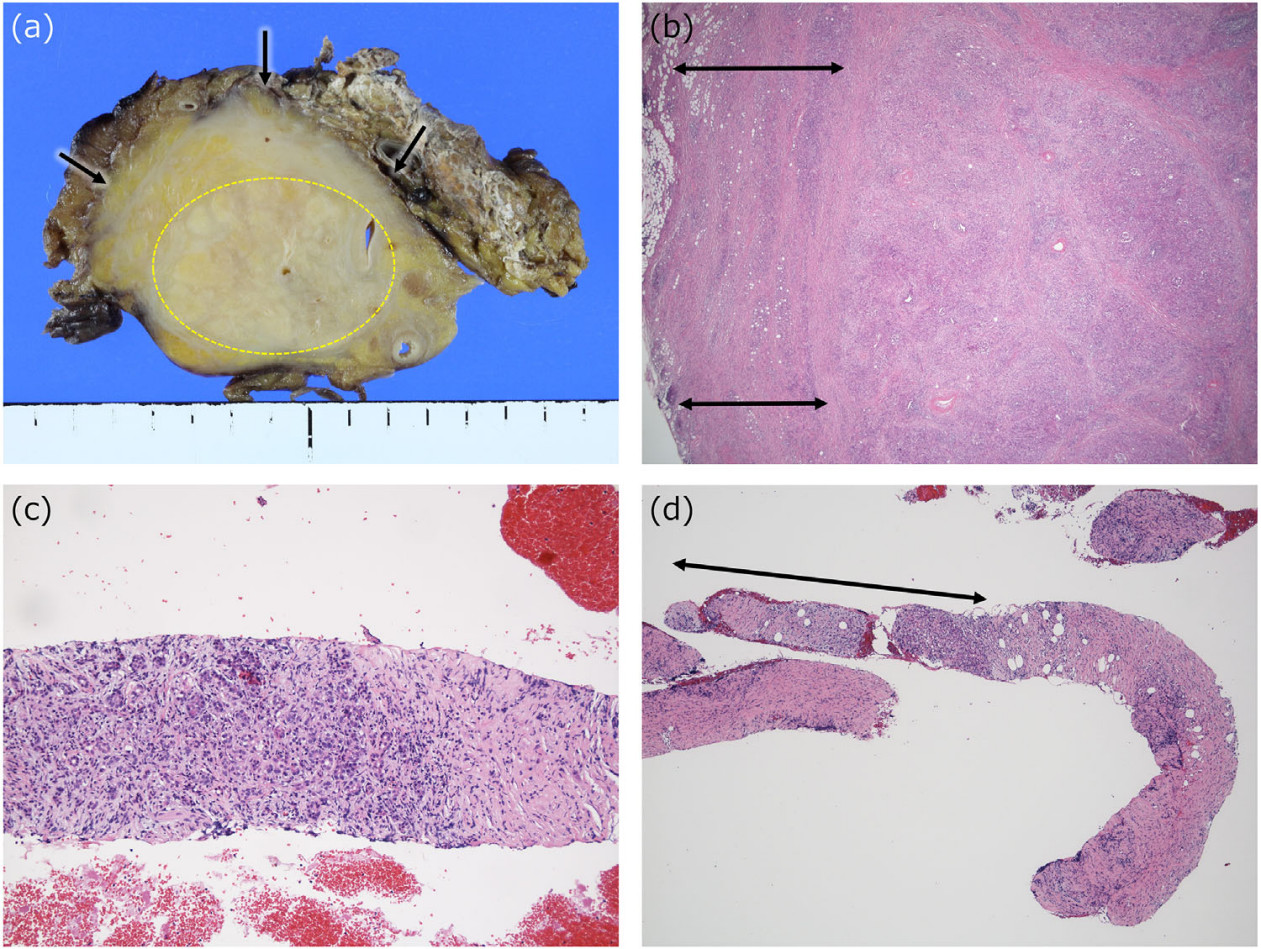
Macroscopic and low‐power histological pictures of resection and biopsy samples of type 1 AIP. In the resected material (a,b), the lobular contours are well preserved (*dotted circle* in (a), and the right two‐thirds in (b)), and the pancreatic parenchyma is surrounded by a capsule‐like rim (*arrows* in (a), *two‐headed arrows* in (b)), which is a collar of an inflammatory lesion. In the biopsy sample (c,d), lobular inflammation bounded by the septum‐like fibrous band (*right side*) is representative (c). When the capsule‐like rim is thick, it can be obtained in a biopsy (the pinkish portion of the tissue; a lobule indicated by a *two‐headed arrow*) (d)

The lesion distribution of type 1 AIP is also unique.^15^ In the biopsy specimens, two components are commonly obtained. The first component is the lobular contours that are well‐preserved and well‐circumscribed despite dense lymphoplasmacytic infiltration (Figure 2a–c). Septum‐like fibrous bands are present between the lobules and make the lobular contours more evident (Figure 2c). The lobular inflammation usually comprises simple lymphoplasmacytic infiltration without otherwise unique features with the exception of the observation of numerous IgG4‐positive cells. This is the reason why the lobular changes have not been emphasized in discussions of the histological features of type 1 AIP. The lobules may be atrophic with fibrosis focally or, on rare occasions, diffusely.

The second component in biopsy specimens of type 1 AIP is the inflammation that is observed in the peripancreatic adipose tissue that is known as a ‘capsule‐like rim’ radiologically (Figure 2a,b).^16^, ^17^ This tissue is where storiform fibrosis and obliterative phlebitis are typically observed in the resected specimens. However, it should be kept in mind that storiform fibrosis is not always present in type 1 AIP lesions. The capsule‐like rim may consist of simple lymphoplasmacytic infiltration (which is difficult to recognize on computed tomography scans in such cases) or almost complete fibrosis in some cases. In addition, the size of the capsule‐like rim varies, and it is often difficult to identify the capsule‐like rim in biopsy samples (Figure 2d).

### Previous studies on biopsy‐based diagnoses of AIP

EUS‐TCB was initially applied for the biopsy diagnosis of AIP in the early 2000s.^18^, ^19^, ^20^ The 19‐gauge (ga.) needle used in EUS‐TCB enabled the acquisition of sufficient tissue for diagnosing AIP, but it is not commercially available now due to the inoperability and the lack of superiority to EUS‐FNA for diagnosing PDAC.^21^ There are reports of the utility of a percutaneous needle biopsy for diagnosing AIP,^22^, ^23^ but this procedure has not been broadly accepted due to an alleged risk of peritoneal seeding in cases with PDAC.^24^, ^25^

In the 2010s, several groups — notably from Japan — attempted to develop a method for making a histological diagnosis of AIP by EUS‐FNA,^26^, ^27^, ^28^, ^29^, ^30^, ^31^, ^32^, ^33^, ^34^ and the use of EUS‐FNB has more recently become common.^35^, ^36^, ^37^, ^38^, ^39^, ^40^ Details of these data have been discussed in systematic reviews and meta‐analyses,^41^, ^42^, ^43^ and I will not repeat the discussion here. Instead, I will focus on the histological diagnoses of the studies, which vary among the reports (Table 1). For example, the rate of the identification of storiform fibrosis varies between 0% and 86%, and that of obliterative phlebitis ranges from 0% to 70%.

**TABLE 1:** Comparison of histological data among reports of the use of EUS‐guided fine‐needle aspiration/biopsy (EUS‐FNA/B) in cases of type 1 autoimmune pancreatitis

		**Needle**								**Diagnosis** **^‡^**	
**First author**	**Year**	**FNA/B**	**FNB needle**	**Gauge (ga.)**	**Patient no**.*	**Fields** **^†^**	**Storiform fibrosis**	**Obliterative phlebitis**	**IgG4 >10**	**Level 1**	**Level 1/2**
Imai	2011	FNA		22	21	0.63 mm^2^ (av.)	0	0	0	0	0
Iwashita	2012	FNA		19	44		38 (86%)	21 (48%)	5 (11%)	19 (43%)	
Kanno	2012	FNA		22	25 (1)	20 (80%)	20 (80%)	10 (40%)	9 (36%)	14 (56%)	20 (80%)
Ishikawa	2012	FNA		22	47 (3)		34 (72%)	0	10/28 (36%)	9 (19%)	14 (30%)
Morishima	2016	FNA		22/25	41		0	0	27 (66%)	0	27 (66%)
Kanno	2016	FNA		22	78	29 (37%)	49 (63%)	38 (49%)	19 (24%)	32 (52%)	45 (58%)
Detlefsen	2017	FNB	FT	22	1		0	1	1	1	1
Sugimoto	2017	FNA		19/22/25	40 (2)					2 (5%)	7 (18%)
Cao	2018	FNA		22	27		18 (67%)	0	8 (30%)	5 (19%)	17 (63%)
Kurita	2020	FNB	F	22	50	23 (42%)	28 (56%)	12 (24%)	38 (76%)	28 (56%)	39 (78%)
		FNB	FB	20	51	8 (16%)	13 (25%)	7 (14%)	22 (43%)	13 (25%)	23 (45%)
Ishikawa	2020	FNB	F	22	55		40 (73%)	24 (44%)	36 (65%)	32 (58%)	51 (93%)
Sugimoto	2020	FNA	(WEST)^§^	19/22	11		5 (45%)	2 (18%)	7 (64%)	4 (36%)	8 (73%)
		FNA	(DRY)^§^	19/22	23		1 (4%)	0	5 (22%)	1 (4%)	3 (13%)
Oppong	2020	FNB	FT	22/25	18		11 (61%)	8 (44%)	14 (78%)	13 (72%)	14 (78%)
		FNB	RB	22	6		0	0	1 (17%)	0	0
Tsutsumi	2021	FNB	Menghini	21	14	6.2 mm^2^ (med.)	5 (36%)	1 (7%)	9 (64%)	5 (36%)	9 (64%)
		FNA		22	14	0.7 mm^2^ (med.)	0	0	0	0	0
Noguchi	2020	FNB	F/RB/FB	22/19/20	32		15%	70%	60%	10 (31%)	7 (22%)
		FNA		19/22/25	21		0	7%	21%	0	4 (19%)

Abbreviations: av., average; EUS, endoscopic ultrasound‐guided; F, Franseen needle; FB, forward‐bevel needle; FNA, fine‐needle aspiration; FNB, fine‐needle biopsy; FT, fork‐tip needle; ga., gauge; med., median; RB, reverse‐bevel needle.

* Reports with ≥3 cases were selected for this table. However, in the Patient no. column, the number of only cases with type 1 AIP are provided. When types 1 and 2 AIP are analyzed together, the number of cases with definite or probable type 2 AIP diagnosis are indicated in the parentheses.

^†^ Number of cases with tissue amounts of >10 high‐power fields (approx. 5 mm) are indicated. Some reports provided the average or median value of tissue dimensions.

^‡^ Levels 1 and 2 here are based on the International Consensus Diagnostic Criteria. Level 1 = three or more items of the histological findings are fulfilled. Level 2 = two of the histological findings are fulfilled.

^§^ Techniques used by the authors. WEST: wet suction technique and the conventional method is indicated as DRY.

These wide variations in data are likely due to the different sample volumes but may also be attributable to different methods used among the studies. Potential reasons are as follows. First, the above‐mentioned features (1) through (4) in the histological findings (Figure 1) were used in most of the studies instead of features (2) through (5), although the studies are based on the ICDC. There is a single study that was based on the original features (2) through (5),^33^ and, as a result, the ratio of cases with the definitive histological diagnosis of type 1 AIP in that study is extremely low (19%, Table 1). Second, IgG4‐positive cells were evaluated in an average of all high‐power fields (HPFs) in some studies,^28^, ^31^ although such cells were counted at hot spots in the other studies.

Third, as indicated by Yoon et al.,^43^ the occurrence of obliterative phlebitis varies greatly between the studies that used elastic stains and those that did not. It is not surprising that obliterative phlebitis is scarcely identified without the help of an elastic stain for the biopsy specimens. Fourth, the concordance of the conclusions of histological evaluations among pathologists might not be sufficient, as will be discussed in detail in a later section. Fifth, in some studies, cases with neither a level‐1 nor level‐2 diagnosis seem to have been excluded with an alternative diagnosis (such as mass‐forming pancreatitis), even though non‐specific findings that make a diagnosis inconclusive can occur in a biopsy from type 1 AIP lesions.^44^ In such a study, the histological diagnostic accuracy might be inflated. Based on all of these inconsistencies, the reported data on the histological findings are not easily compared.

### Merits and limitations of diagnosing type 1 AIP when large biopsy specimens are obtained

**FIGURE 3:**
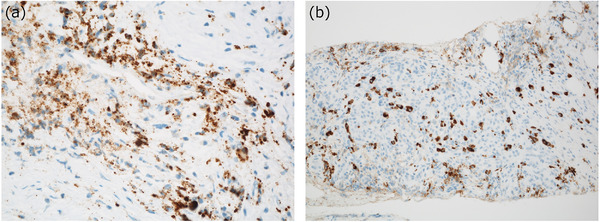
Immunoglobulin G4 (IgG4)‐immunostaining in the biopsy samples of type 1 autoimmune pancreatitis (AIP). In a small sample, immunostaining often fails (a). This is due to the staining of fragmented cytoplasm of plasma cells caused by a crushing artifact. In a large specimen, immunostaining for IgG4 is often easy to evaluate (b)

To clarify the merits and limitations of the pathological diagnosis when large tissue samples are acquired by EUS‐FNB, our research group carried out a nationwide multicenter study in Japan which we named the ‘guidance project’ because our goal was to establish guidelines for diagnosing type 1 AIP with biopsy specimens based on the data analyzed. We attempted to collect tissue samples obtained by EUS‐FNA/B that were as large as possible (including cases with PDAC) from each institute.^45^ In this manner, we sought to clarify how often the pathological diagnosis of type 1 AIP could be rendered, what the issues were, and whether the diagnosis was concordant among pathologists when sufficient amounts of biopsy samples were obtained. The results of our analysis of the collected non‐neoplastic tissues (*n* = 85, including 86% of cases with definite type 1 AIP) indicated that IgG4 immunostaining was successful in most of the cases; the exceptions were the cases with smaller amounts of tissue (<2.5 mm in length) (Figure 3).^45^ We also observed that the cytoplasmic fragmentation of plasma cells was marked in smaller tissue samples and caused background staining that made the immunohistochemical evaluation difficult (Figure 3a). Thus, successful immunostaining for IgG4 seems to be the greatest advantage of obtaining large biopsy samples (Figure 3b). This trend was also suggested by Kurita et al., who observed >10 IgG4‐positive cells more frequently in cases obtained with a 22‐ga. Franseen needle compared to those obtained with a 20‐ga. forward‐bevel needle, and the sample sizes were significantly larger in the former type of cases.^36^ The sample size is also important because smaller samples are often severely crushed, and it is difficult to evaluate the histological features due to the crush artifacts.

In our above‐cited nationwide multicenter study, the rates of the presence of storiform fibrosis, obliterative phlebitis, and the ductal lesion were 37.6%, 28.2%, and 22.4%, respectively; not surprisingly, tissues with each finding were significantly larger than those without, suggesting that large tissue samples are necessary for identifying these findings.^45^ However, when tissues <5 mm in length were excluded, the statistical significance remained for obliterative phlebitis and the ductal lesion, but not for storiform fibrosis. These data indicated that storiform fibrosis might be completely absent in some type 1 AIP cases.

As indicated here, the acquisition of sufficient amounts of tissue is important for the biopsy diagnosis of type 1 AIP.^46^ Data analyses of the biopsy tissues from type 1 AIP lesions should be based on the sample volumes, and not a simple comparison between FNA and FNB, as one research group obtained adequate specimens for the diagnosis even by EUS‐FNA.^28^

### Can pathologists diagnose type 1 AIP consistently with biopsy samples?

The degree of agreement between pairs of pathologists has been studied, and substantial to an almost‐perfect agreement regarding four histological features was reported.^28^, ^36^ We carried out such a study of 20 participating pathologists (nine specialists who were familiar with pancreatic pathology or IgG4‐related disease and 11 general pathologists) in the guidance project. The results were suboptimal; slight‐to‐moderate agreements among the observers on storiform fibrosis, obliterative phlebitis, and the ductal lesion.^47^ Specifically, the agreement regarding obliterative phlebitis was the best (moderate agreement), and the agreement about the ductal lesion among the generalists was the worst (slight agreement).

**FIGURE 4:**
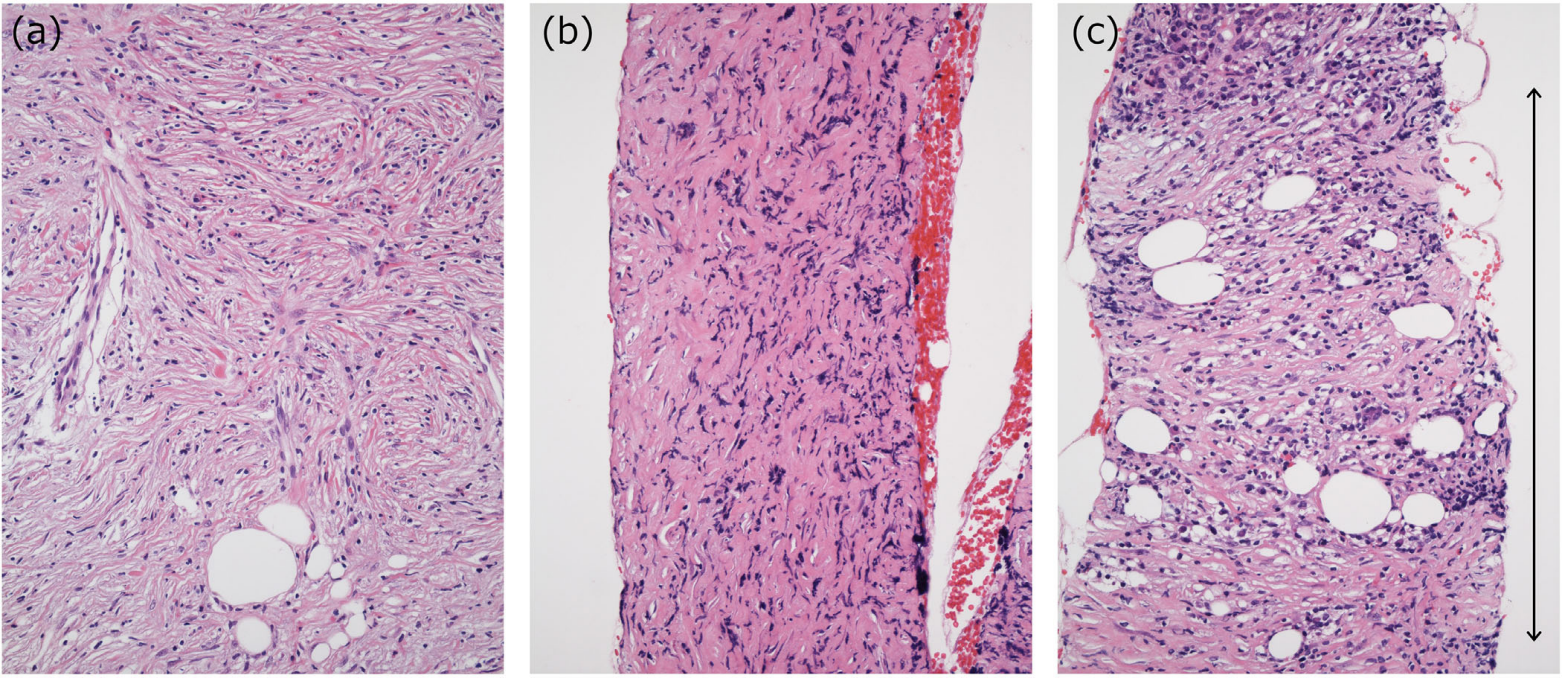
Storiform fibrosis and a mimicker in biopsy samples of type 1 autoimmune pancreatitis (AIP). (a) Typical storiform fibrosis. (b) Marked fibrosis seen in a portion of the capsule‐like rim. The tissue may resemble storiform fibrosis by the crushing artifact, but it is not storiform fibrosis. (c) A right‐angular sampling of a flowing arrangement of storiform fibrosis (*two‐headed arrow*) may be difficult to identify because the entire pattern cannot be evaluated in a biopsy tissue sample

There are several reasons for the potential disagreements in histological evaluations of type 1 AIP in biopsy samples. The first is the lesion heterogeneity depending on the time phases of the inflammation. Type 1 AIP is well known to regress spontaneously and therefore reveals heterogeneous histological profiles depending on the activities. For example, storiform fibrosis gives rise to features that resemble the non‐specific fibrosis observed in chronic inflammations when the number of inflammatory cells decreases (Figure 4b). For some lesions, it might even be difficult to diagnose whether storiform fibrosis is present or not. In resected specimens, there might be foci that are typical of storiform fibrosis even in lesions that are otherwise markedly regressed, but such an approach is not possible with a biopsy.

The second possible reason for the disagreement among pathologists is the appearance of only a portion of the characteristic findings in the biopsy specimens. A portion of obliterative phlebitis or the ductal lesion present at the edge of biopsy tissues, which is not uncommon, is difficult to identify. A flowing arrangement of storiform fibrosis may also be difficult to identify when the lesion is biopsied at a right angle (Figure 4c).

**FIGURE 5:**
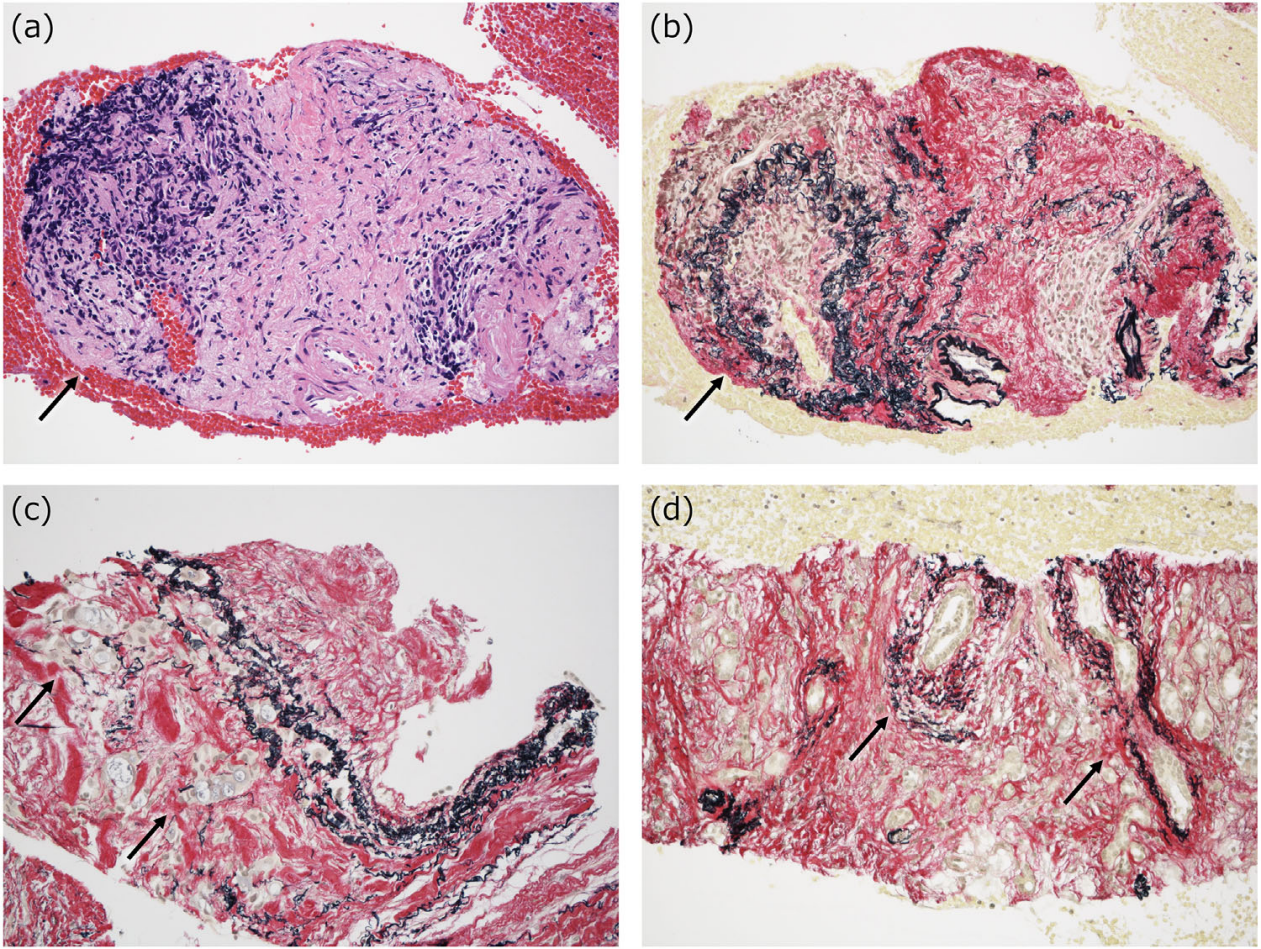
Obliterative phlebitis of type 1 autoimmune pancreatitis (AIP) and mimickers seen in the biopsy samples. (a,b) Obliterative phlebitis (*arrows*) is difficult to identify on the hematoxylin‐eosin (HE)‐stained slides (a), and the assistance of the elastic stain (Verhoeff Van Gieson [VVG] stain in this case) is necessary (b). (c) Fibrous venous occlusion can be identified in pancreatic ductal adenocarcinoma (PDAC) with VVG stain. Note that cancer cells are present (*arrows*). (d) Elastic fibers are also present in the connective tissue, notably around the pancreatic ducts (*arrows*), which may resemble obliterative phlebitis ((d); VVG stain)

The third reason for disagreement in histological evaluations is the necessity of using elastic stains for obliterative phlebitis because this method has some pitfalls. In resected specimens, obliterative phlebitis can be identified on hematoxylin‐eosin (HE)‐stained slides by identifying a replacement of pre‐existing venules by fibroinflammatory nodules.^1^ However, in biopsy specimens, only smaller venules that are difficult to identify on HE‐stained slides alone are usually obtained, and an elastic stain is necessary to identify obliterative phlebitis (Figure 5a,b). This method also depicts the fibrous venous occlusions that are observed, for example, in chronic pancreatitis and PDAC that are not identifiable on HE‐stained slides (Figure 5c).^45^, ^48^ It is also important to remember that elastic fibers are observed in the normal connective tissues, notably around the pancreatic ducts (Figure 5d), and that these fibers are another mimicker of obliterative phlebitis.

Finally, as mentioned earlier, the definitions of the histological features, notably of storiform fibrosis, can differ among pathologists.

### Guidance for diagnosing type 1 AIP with biopsy tissues

Based on all of these potential issues regarding the biopsy diagnosis of type 1 AIP, we have developed guidance for diagnosing type 1 AIP with biopsy tissues with the participation of Japanese gastroenterologists and pathologists.^49^ This guidance is completely based on the JPS2018,^12^ and the features of the histological findings are unchanged. In other words, we are not proposing new criteria; rather, a detailed explanation of the histological features is given in this guidance. This is why the name “guidance” was adopted. To establish the precise diagnosis of type 1 AIP, the guidance emphasizes the specifics of each histological feature.

#### IgG4‐positive cells

The cutoff value of the number of IgG4‐positive cells for diagnosing type 1 AIP is difficult to determine. It is well known that IgG4‐positive cells >10/HPF, which is used as the cutoff value of numerous IgG4‐positive cells in the ICDC and JPS2018, are not specific for type 1 AIP and other IgG4‐related diseases. The new guidance thus provides additional information to make the evaluation of IgG4 immunostaining more specific. The guidance notes that a diffuse infiltration of IgG4‐positive cells and an increased IgG4/IgG‐positive ratio (>40%)^50^ are also features of AIP. It is difficult indeed to detect a diffuse distribution of positive cells in a biopsy sample, and the guidance recommends the identification of multiple foci with numerous IgG4‐positive cells as an alternative way to evaluate the sample. When these additional features are absent, the diagnosis of type 1 AIP must be made cautiously.

#### Storiform fibrosis

**FIGURE 6:**
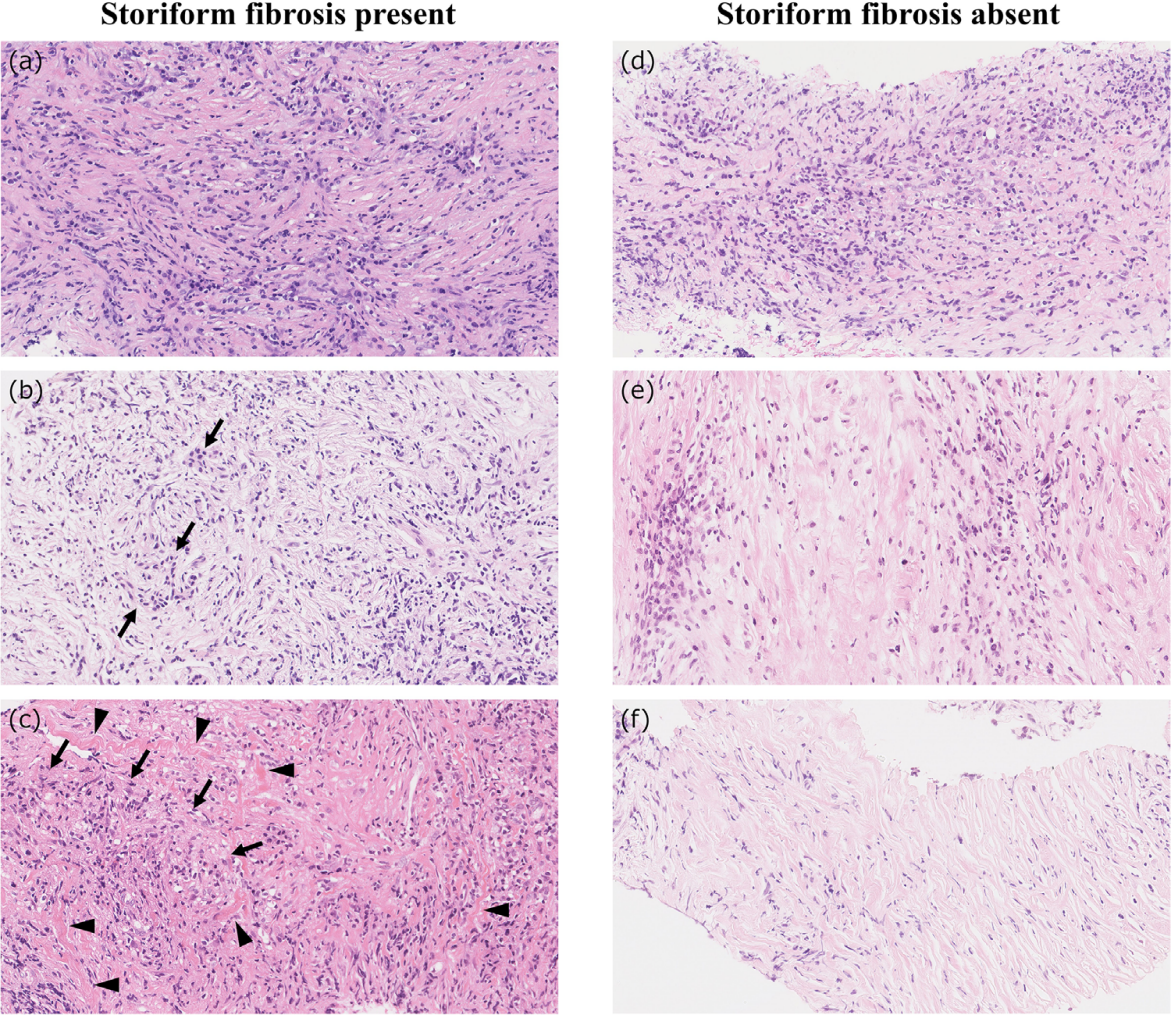
Examples of the presence (a–c) and absence (d–f) of storiform fibrosis, which are included in the guidance (reuse of the picture in the guidance^49^ with permission). (a–c) Storiform fibrosis shows a flowing arrangement of spindle‐shaped cells, inflammatory cells, and delicate collagen as a unit (a,b). Although thick collagen strands (*arrowheads*) are atypical, a portion with the typical features (*arrows*) allows the diagnosis of storiform fibrosis (c). (d–f) Examples of samples that are insufficient to diagnose storiform fibrosis. Although inflammatory cells are numerous (d) or both inflammatory cells and delicate fibrosis are present (e), a flowing arrangement is absent. A flowing arrangement of collagen fibers is present, but inflammatory cells are scarce (f). However, these findings do not exclude a diagnosis of type 1 autoimmune pancreatitis (AIP)

Storiform fibrosis is defined as a flowing arrangement of cell‐rich components that include spindle‐shaped cells and inflammatory cells in the background of delicate collagen. As (mentioned above) ambiguous lesions are present, the new guidance provides photographs with storiform fibrosis that is present or absent, based on a consensus among the five working group members (Figure 6).

#### Obliterative phlebitis

Obliterative phlebitis is defined as an obstructive or stenotic venous lesion that is caused by the characteristic inflammatory changes of type 1 AIP. Both fibrosis and inflammatory cell infiltration are necessary for the obliterated lesions to distinguish obliterative phlebitis in type 1 AIP from the fibrous venous occlusions (Figure 5a,b). Importantly, as mentioned earlier, the use of elastic stains is necessary to identify obliterative phlebitis in biopsy specimens.

#### Other histological findings

**FIGURE 7:**
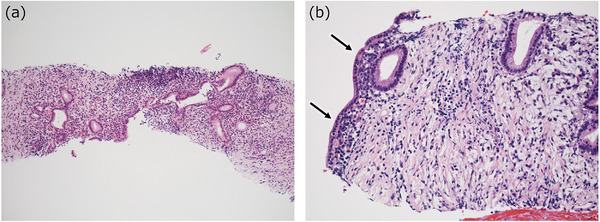
The ductal lesion of type 1 autoimmune pancreatitis (AIP) present in a biopsy sample. A typical example of the ductal lesion, which is infrequently encountered in biopsy specimens ((a); reuse of the picture in the guidance^49^ with permission). The ductal lesion may be present at the edge of a biopsy sample (b). Note that the epithelium (*arrows*) is surrounded by a cuff of inflammatory cells

The guidance explains that the ductal lesion, periarteritis, and perineurial inflammation may be encountered in type 1 AIP. The ductal lesion of type 1 AIP is a collar of inflammatory cell infiltration that surrounds the duct epithelium without damaging the epithelium itself (Figure 7a). The ductal epithelium is often present at the edge of a biopsy specimen (Figure 7b).

Notably, a distinction of the ductal lesion of type 1 AIP from lymphoplasmacytic infiltration that is adjacent to but does not involve the duct itself is difficult because the former never damages the epithelium. Thus, among the histological features, the level of agreement among the pathologists about the ductal lesion was the worst.

### The guidance's effect on the concordance among observers

After showing the new guidance to the observers, we carried out round 2 of the agreement study.^47^ The κ‐value among the specialists for storiform fibrosis increased from 0.231 to 0.262. In contrast, the κ‐value for obliterative phlebitis decreased from 0.457 to 0.327 among the specialists and from 0.537 to 0.374 among the generalists, which was probably due to the lack of familiarity with distinguishing obliterative phlebitis from fibrous venous occlusions among the observers. Even so, we maintain that the descriptions of obliterative phlebitis are important, and we will modify them in the future to achieve better concordance in keeping with the current descriptions. Regarding the ductal lesion, no improvement in the agreement was achieved with the guidance. Considering that the diagnosis is difficult, we suggest that the ductal lesion is not suitable as a key finding in biopsy specimens.

### The pathology‐based distinction between PDAC and type 1 AIP in biopsy specimens

The results of cytological examinations of the pancreatic juice and pancreatic duct brushing are sometimes difficult for cytopathologists to evaluate, due to the necessity of distinguishing cancerous cells from various types of premalignant cells or regenerative cells in the duct. In contrast, specimens of PDAC obtained by EUS‐FNA/B consist only of invasive cancerous cells with no contamination of other types of atypical cells, and they are thus believed to be easier for cytopathologists to use successfully. However, this is not always the case; this is notably a problem in type 1 AIP cases because one purpose for acquiring tissues from patients with possible type 1 AIP is to exclude PDAC.

We examined the interobserver agreement for distinguishing PDAC and non‐neoplastic lesions (definite type 1 AIP in 86% of the cases) in the guidance project.^47^ Seven pathologists who were familiar with pancreatic pathology and 13 general pathologists were enrolled. The κ‐values were 0.886 (almost perfect agreement) among the specialists and 0.750 (substantial agreement) among the generalists. However, cases with misinterpretation did exist, and a post‐investigative questionnaire indicated that acinar‐ductal metaplasia (ADM)^51^ was confusing for all of the participants. In addition, the generalists found it difficult to evaluate minimal atypia of PDAC.

**FIGURE 8:**
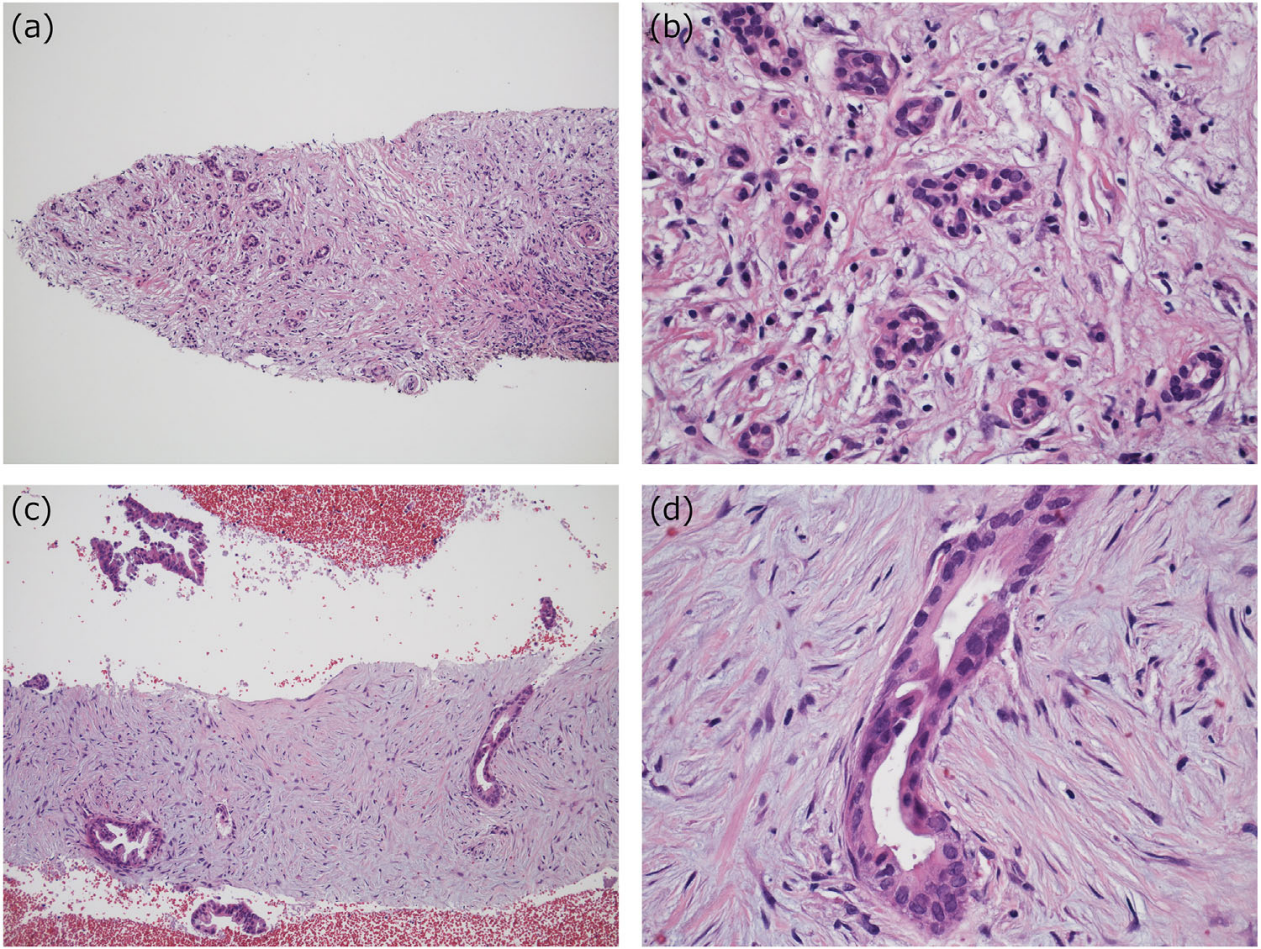
Comparison of acinar‐ductal metaplasia (ADM) and pancreatic ductal adenocarcinoma (PDAC). (a,b) ADM present in a biopsy sample of type 1 autoimmune pancreatitis (AIP). Glands are present focally within a lobule (a). Glandular lumens are indistinct, and the cells have small round nuclei (b). (c,d) Glands of PDAC in a biopsy sample. Glands are distinct, and clusters of cancer cells are also commonly present around the core tissue (c). Although PDAC cells are often indolent, the nuclei are even larger, and they are more irregular in size and shape (d) compared to those in ADM (b). Note that the stromal cells forming a desmoplastic reaction (d) are plumper than those in type 1 AIP (b)

ADM is an increase of duct‐like structures within the pancreatic lobules, and this probably represents a metaplastic process of acinar cells (Figure 8a,b).^49^ The glands observed in ADM have mostly indistinct glandular and luminal shapes, and they are often difficult to detect. However, distinct glands are sometimes encountered, which thereby causes a problem in distinguishing them from glands of PDAC (Figure 8c,d). This is particularly true in type 1 AIP because the background stroma consisting of inflammatory cell infiltration and fibrosis resembles those in PDAC.

The guidance delineates what ADM is and explains how to distinguish ADM from PDAC. In round 2 of the agreement study,^47^ which was performed after the observers read the guidance, the κ‐values increased to 0.958 and 0.815 (both almost perfect agreement) for the specialists and generalists, respectively, suggesting that the guidance is effective for achieving an accurate distinction of PDAC and type 1 AIP.

### Closing remarks

Although a histological diagnosis of type 1 AIP with biopsied specimens obtained by EUS‐FNB is feasible, the relevant agreement among pathologists about the diagnosis is suboptimal. Moreover, the distinction between type 1 AIP and PDAC may be difficult in some cases. In order to cope with the issues raised in this review article, a multi‐disciplinary approach with the collaboration of clinicians, radiologists, and pathologists is necessary to prevent confusion.

## CONFLICT OF INTEREST

The author declares no conflict of interest. This is a review article, and approval by the ethical review board is not required.

## FUNDING INFORMATION

This work was supported by MHLW's research program on rare and intractable diseases (Grant Number: JPMH20FC1040).
